# Results of a Pilot Trial Assessing the Effects of Proper Oral Hygiene and a Probiotic Dietary Supplement on Oral Health in Volunteers with Oral Malodor

**DOI:** 10.3390/microorganisms12091821

**Published:** 2024-09-03

**Authors:** Elena Y. Enioutina, R. James Keddington, Kurtis G. Hauck, Amarina Chavez, Jeffrey J. Clifford, Thy (April) Cao, Bryce Smith, Kathleen M. Job, Alfred Balch

**Affiliations:** 1 The Division of Clinical Pharmacology, Pediatrics, School of Medicine, University of Utah, Salt Lake City, UT 84108, USA; kate.job@hsc.utah.edu; 2 School of Dentistry, University of Utah, Salt Lake City, UT 84108, USA; james.keddington@hsc.utah.edu (R.J.K.); hauckkurtis@gmail.com (K.G.H.); amarinachavez@gmail.com (A.C.); 3 The Department of Pathology, School of Medicine, University of Utah, Salt Lake City, UT 84112, USA; jeff.clifford@path.utah.edu (J.J.C.); april.cao@hsc.utah.edu (T.C.); bryce.smith@utah.edu (B.S.); 4 Family and Preventive Medicine Department, School of Medicine, University of Utah, Salt Lake City, UT 84108, USA; alfred.balch@hsc.utah.edu

**Keywords:** probiotics, oral health, proper dental hygiene, malodor, tongue coating, salivary cytokines, probiotic DNA levels, pilot trial

## Abstract

Persistent malodor affects many people worldwide and is usually associated with poor dental hygiene. This pilot trial aimed to determine whether proper dental hygiene (DH) and a probiotic dietary supplement support oral health in volunteers with persistent malodor. Volunteers (*n* = 35) were randomly assigned to the probiotic or placebo cohort. The probiotic cohort (*n* = 20) brushed and flossed their teeth twice daily and used probiotics for 30 days; the placebo cohort (*n* = 15) followed the same hygiene practices and used the placebo. The intervention phase was followed by a 30-day follow-up period. Measured outcomes were malodor and tongue-coating scores, probiotic DNA levels, salivary cytokines, and salivary pH. DH and probiotics significantly decreased malodor (~50% during intervention) and tongue coating scores (~45% during intervention). These changes remained through the course of the trial. The probiotic DNA levels increased in the probiotic cohort and dropped in the placebo cohort after the intervention started. The malodor moderately correlated with the tongue coating *P. acidilactici* level. The addition of probiotics increased IL-10 levels during the intervention and decreased IL-8, TNF-α, and IL-6 by the end of the study. People with malodor may benefit from using DH and probiotics. Additional trials are needed to definitively establish the benefits of probiotic dietary supplements.

## 1. Introduction

A malodor (i.e., halitosis) is usually described as an unpleasant odor coming from a person’s mouth “with a severity exceeding a socially acceptable level” [1,2,3]. The etiology of malodor could be intraoral or extraoral. Eighty to ninety percent of malodor is associated with intraoral factors such as poor dental hygiene (DH), heavy tongue coating (physiological halitosis), periodontal diseases, Sjogren syndrome, and cancer treatment (pathological halitosis) [1,4]. Contributing factors of the physiological malodor could be wide interdental spaces and food retention between teeth, orthodontic appliances, and tongue biofilm [4]. In comparison, extraoral factors leading to malodor are systemic diseases (e.g., sinusitis, tonsilitis, hepatic failure, gastro-esophageal reflux, gastritis caused by *Helicobacter pylori* infection, diabetic ketoacidosis, and many others) [4,5]. The common cause of malodor is bacteria producing odoriferous components such as volatile sulfur components and diamines (e.g., *Actinomyces* spp., *Dialister* spp., *Eubacterium* spp., *Fusobacterium* spp., *Bacteroides* spp., and *Peptostreptococcus* spp.) [5,6].

Because of the socially sensitive issue of malodor, there is limited data on the epidemiology of malodor. According to Bollen and Beikler, bad breath has “a large social and economic impact” [7]. Several studies reported a malodor prevalence of up to 23% in China, 50% in the US, and up to 35% in India [4]. Increased oral malodor could also be more prevalent in older populations.

In addition to the unpleasant odor and tongue coating, cytokines can serve as biomarkers of oral health and periodontal diseases [8]. Individuals with good oral health have high levels of salivary TNF-α, IL-17, and IL-10, while individuals with periodontal diseases have increased levels of IL-1β and IL-6.

The general public often associates oral health with the absence of an unpleasant odor from the mouth and minimal or no tongue coating. Almost all people experience a transient unpleasant odor coming from the mouth. However, up to 50% of individuals living in developed countries report persistent episodes of oral malodor [6,9,10,11]. Unfortunately, many people worldwide fail to brush their teeth daily, leading to malodor and asymptomatic suboptimal health of the gingivae [12].

Multiple interventions are proposed to reduce/eliminate malodor. Among them are professional dental cleaning, antimicrobial toothpaste, mouthwashes, pine bark extract chewing gums, or probiotics [5,13,14,15]. Most studies investigating probiotics’ effect on oral health reported an improvement in overall oral health, a decrease in unpleasant odor, and a decrease in the tongue coating index [14,16]. However, a systemic review and meta-analysis of the effects of probiotics on malodor concluded that probiotics, mainly *Lactobacillus* strains, had a moderate impact on organoleptic scores [16].

This pilot randomized clinical trial was aimed to determine whether proper DH (teeth brushing and flossing twice a day) and the use of a dietary supplement containing three freeze-dried lactic acid bacteria strains will reduce malodor and tongue coating scores, affect salivary pH, and modulate pro- and anti-inflammatory cytokines in people with persistent malodor.

## 2. Materials and Methods

### 2.1. Participants and Inclusion/Exclusion Criteria

Thirty-five healthy adult volunteers (males and females, 18–55 years old) with a persistent malodor were enrolled in the study. The participants who participated in the trial were unaware whether they were assigned to a placebo or probiotic cohort.

The following individuals were included in the trial:

1. Individuals that were 18–55 years old;
2. Individuals with a persistent malodor (≥3 months);
3. Individuals with organoleptic scores 2–5.

The following individuals were excluded from the trial:

1. Individuals who were less than 18 years of age or over 55 years of age at enrollment;
2. Individuals who had minor persistent malodor scores (scores 0–1);
3. Individuals who had dentures;
4. Individuals who reported a professional dental cleaning within 1 month of randomization;
5. Individuals who reported any gastrointestinal (Reflux esophagitis, gastritis, ulcer, hiatal hernia, Zencker diverticulum, achalasia) and ENT disorders (pharyngitis, tonsillitis, sinusitis, bronchiectasis);
6. Female individuals who reported being pregnant or intending to become pregnant in the next two months;
7. Individuals who reported the use of immunosuppressive therapy;
8. Individuals who reported immune deficiencies.

All eligible participants signed a consent form to participate in the trial and were randomized into 5:5 blocks using randomizer.org.

### 2.2. Study Design

This pilot parallel, single-blind, randomized, placebo-controlled trial was conducted at the University of Utah during the years of 2021–2023. Ethics approval was obtained from the University of Utah Institutional Review Board (IRB, No. 00131500). Participants signed an informed consent form approved by the IRB. The study was designed bearing in mind that general consumers with persistent malodor might try to improve their oral health by using proper DH (brushing and flossing their teeth twice daily) and oral probiotic lozenges. Brushing and flossing techniques were adopted by the American Dental Association. The study was advertised at the University of Utah Information and Advertisement boards and the University of Utah student dormitories/University Village.

All participants were provided the same soft toothbrushes and toothpaste for sensitive teeth. The participants were asked to use DH during the first 30 days of the trial. The participants in the probiotic cohort (*n* = 20) were instructed to use one lozenge of probiotics in the morning after brushing and flossing teeth and one lozenge in the evening after brushing and flossing teeth. The placebo cohort received similar instructions on brushing and flossing teeth and the use of placebo lozenges (*n* = 15). Participants were recommended to refrain from eating food and drinking any liquid for at least 1 h after using the lozenge. Additionally, participants received instructions to refrain from chewing gum and tongue cleaning.

The 30-day intervention phase was followed by a 30-day follow-up period. The participants attended office visit 1 (baseline), visit 2 (intervention), and visit 3 (follow-up), where participants were evaluated for the intensity of malodor and tongue coating. Salivary samples were collected for the evaluation of cytokine levels. The tongue coating was evaluated for the DNA levels of probiotic stains.

At office visit 2, participants were asked whether they brushed and flossed their teeth and took lozenges as recommended. All participants were asked to bring lozenge blisters to office visit 2 to evaluate compliance with the protocol.

#### 2.2.1. Office Visits Activities

In the dental office of the University of Utah School of Dentistry, the participants were evaluated for malodor scores and tongue coating index during each office visit. The participants were also asked to collect saliva and tongue-coating samples for further evaluation.

**Figure 1:**
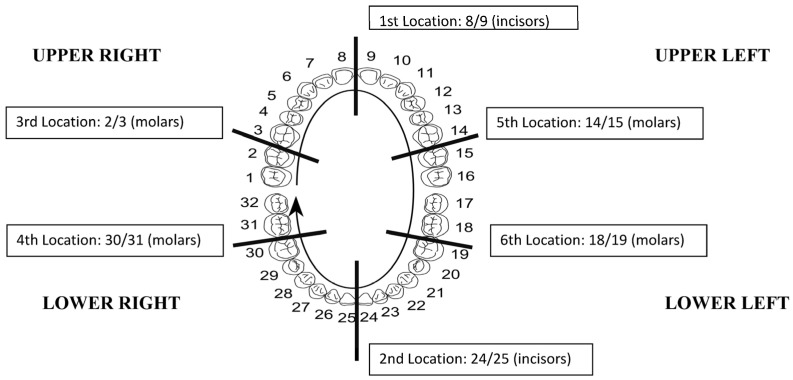
In-between teeth positions at which participants evaluated the intensity of malodor. Modified from https://en.wikipedia.org/wiki/Universal_Numbering_System (accessed on 20 May 2024). The Universal Numbering System image was created by Kaligula (http://commons.wikimedia.org/wiki/User:Kaligula) accessed on 11 May 2013.

The level of oral malodor was evaluated on a 0 to 5 scale (organoleptic scale) as follows: 0—absence of odor, 1—questionable odor, 2—slight malodor, 3—moderate malodor, 4—strong malodor, and 5—severe malodor [17]. Participants measured organoleptic scores between teeth in six different areas during office visits based on the organoleptic scale (Figure 1). Participants winded 12–18 inches of floss around the index finger of one hand and the middle of the other hand, maintaining a two-inch length of floss stretched between fingers. Then, participants placed the dental floss between teeth, gently sliding it up and down and rubbing it against the sides of each tooth. After removing the floss, participants immediately sniffed and scored the floss odor based on the organoleptic scale.

**Figure 2:**
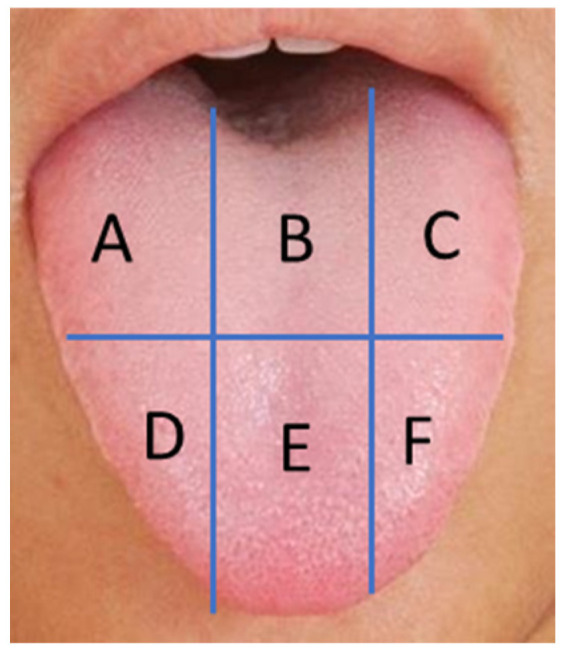
Tongue division into six areas for tongue coating evaluation. A–F tongue areas where the coating scores were evaluated.

The investigators assessed the tongue coating using the Winkel tongue coating index [18]. The dorsum of the tongue was divided into six areas—three in the anterior and three in the posterior part (Figure 2). Tongue coating was assessed in six areas using the following scale: 0 = no coating, 1 = slight coating, and 2 = heavy coating. After a subjective evaluation of the tongue coating, the participants gently scraped their tongues with a single-use Philips Sonicare BreathRxtongue cleaner (Andover, MA, USA) and placed the scrapings into cryovials. Samples were stored at −80 °C before analysis.

Additionally, participants were asked to collect an ~1-milliliter saliva sample by passively drooling into two labeled cryovials, amounting to 0.5 mL in each vial. Samples were stored at −80 °C until shipment to Salimentrics (Carlsbad, CA, USA) for cytokine analysis.

#### 2.2.2. At-Home Malodor Measurements

Participants were asked to measure organoleptic scores every seven days while at home. These measurements were performed in the morning before breakfast, tooth brushing, and tooth flossing.

### 2.3. Probiotic and Placebo Lozenges

The probiotic lozenges used in the study are a dietary supplement composed of a freeze-dried lactic acid bacteria 1:1:1 blend of 3 strains (*Lactobacillus brevis* KABP052 (CECT 7480), *Lactobacillus plantarum* KABP051(CECT 7481), and *Pediococcus acidilactici* KABP053 (CECT 8633) in a base of sorbitol, guar gum, mint flavor, and magnesium stearate. The lozenges contained > 1 billion CFUs of a total of three strains. Placebo lozenges contained sorbitol, guar gum, mint flavor, and magnesium stearate.

All lozenges were stored in a −20 °C freezer before they were dispensed to participants. The lozenges were analyzed for their total probiotic microbial count throughout the study period, and the potency met >1 billion CFUs consistently.

### 2.4. Outcomes

Since the probiotic lozenges were used as a dietary supplement, the primary outcomes of this trial focused on evaluating the following body functions:

1. The intensity of oral odor based on the mean organoleptic scores at six interdental positions (Figure 1);
2. The tongue coating scores measured at 6 different locations (Figure 2).

Secondary endpoints included the following:

1. Salivary inflammatory/anti-inflammatory cytokine levels;
2. The DNA levels of the probiotic strains in the tongue coatings by Quantitative PCR (qPCR) analysis;
3. Salivary pH.

### 2.5. Probiotic DNA Levels Analysis

The purpose of analyzing the presence of probiotic strains in the tongue coatings of the participants was to determine at what levels the probiotics would be present in the tongue coating of the participants after the intervention phase and whether they would still be present in the tongue coatings at the end of the follow-up period.

Tongue coating samples were held at −80 °C until testing. Bacterial DNA was extracted with the Maxwell^®^ 16 Buccal Swab LEV DNA Purification Kit (Promega, Madison, WI, USA) and the Maxwell^®^ 16 instrument (Promega, Madison, WI, USA) using a modified protocol wherein tongue coating samples were added to the clearing column and centrifuged after the addition of the lysis solution and incubation at 56 °C. The final eluted volume of DNA was 50 µL.

Real-time PCR was performed using the Lightcycler 2.0 instrument (Roche, Mannheim, Germany), which uses 20 µL glass capillaries. Interactions between glass capillaries and master mix components required the addition of bovine serum albumin (BSA) to the master mix, with a final concentration of 0.5 mg/mL of BSA. Each reaction had 18 µL of master mix and 2 µL of extracted, undiluted sample bacterial DNA. Samples were tested in separate capillaries for *L. plantarum* KABP051, *L. brevis* KABP052, *P. acidilactici* KABP053, and 16S. The PCR cycling protocol was as follows: 1 min at 50 °C for UDG activation, 2 min at 95 °C for the activation of Dual-lock DNA polymerase, followed by 40 cycles of 95 °C for 15 s and 60 °C for 60 s for DNA amplification. Crossing threshold data were obtained using the fit points method of the absolute quantification analysis tool in Lightcycler software 5.0.

Primer oligonucleotides were synthesized by the DNA/Peptide Facility, part of the Health Sciences Center Cores at the University of Utah. Primers were resuspended in MBG water to a concentration of 100 µM. The primer sequence was provided by Kaneka Probiotics USA, CA, USA. Primer sequences are as follows:

*L. plantarum* KABP051 forward 5′-AGGGTTGGACGAGACAA-3′;
*L. plantarum* KABP051 reverse 5′-CCAAACTCATCGGACCTATTC-3′;
*L. brevis* KABP052 forward 5′-TTAGCGTCGTTAGTTGTTATAGG-3′;
*L. brevis* KABP052 reverse 5′-ACGTTCTTGGTCATCGTAATC-3′;
*P. acidilactici* KABP053 forward 5′-AGCTACAGCTACCCAATCT-3′;
*P. acidilactici* KABP053 reverse 5′-GTCGGTTGTGTCCATTAAGT-3′.

The following universal 16S bacterial primers were used as follows [19]:

Forward Primer BACT1369F CGGTGAATACGTTCYCGG;
Reverse Primer PROK1492R GGWTACCTTGTTACGACTT.

Calculation of copy numbers. The standard curves were made by first measuring the A260 of DNA extracted from pure samples of each probiotic species. The mass of DNA in each standard was calculated using the measured absorbance values. The genome copies of DNA per microliter in the standard were calculated based on the genome length of each species and the mass of DNA in the standard. PCR on each standard dilution series was run in duplicate to create a standard curve correlating PCR cycle threshold results with copies of DNA/μL of a sample. The standard curves were made linear by graphing the cycle threshold on the *y*-axis and logging the value of copies of DNA per μL on the *x*-axis. The following formula was used to calculate copies of DNA per μL of the extracted sample: X = 10^((CT-b)/m), where CT is the cycle threshold, b is the y-intercept of the standard curve, and m is the slope of the standard curve (Table 1).

### 2.6. Salivary Cytokines

Salivary samples were analyzed in duplicate for the levels of IL-1α, IL-6, IL-8, TNF-α, and IL-10 using Luminex technology. The analysis was performed by Salimetrics (Carlsbad, CA, USA).

### 2.7. Salivary pH

Before samples were frozen, salivary pH was measured using Ultra-Clear Wide-Range pH Test Strips (Bartovation, New York, NY, USA). Test strips were dipped into saliva for 15 s, and strip color were evaluated against standard colors.

### 2.8. Statistical Analysis

Data were stored in a HIPAA-compliant fashion in the University of Utah Health Sciences Redcap™ database and were extracted and analyzed using the R^®^ language version 3.6.3 (29 February 2020), Copyright (C) 2020 The R Foundation for Statistical Computing and the RStudio interface © 2009–2022, Posit PBC (Boston, MA, USA).

#### 2.8.1. Summary Statistics

Summary statistics for all endpoints (patient demographics, malodor scores, the tongue coating index, probiotics DNA levels in the tongue coating, and salivary cytokine levels) were provided by office visits and home measurements for two cohorts of participants.

#### 2.8.2. Malodor, Tongue Coating, and Cytokine Concentration Analysis

A simple linear mixed model with a random subject effect was used to assess the difference in trend for malodor, tongue coating, and salivary cytokine concentration in the placebo vs. probiotic cohorts. We evaluated whether the change from baseline differed between the two intervention groups at a particular visit using a student *t*-test on the estimated regression coefficient comparing the change from baseline for placebo and probiotic groups, representing the time-by-intervention interaction at a specific time point.

#### 2.8.3. PCR Data Analysis

A 2-sided Wilcoxon rank-sum test was used to compare placebo- and probiotic cohorts’ quantitative DNA levels for *L. plantarum*, *L. brevis*, and *P. acidilactici*.

## 3. Results

### 3.1. General Characteristics of Participants

**Figure 3:**
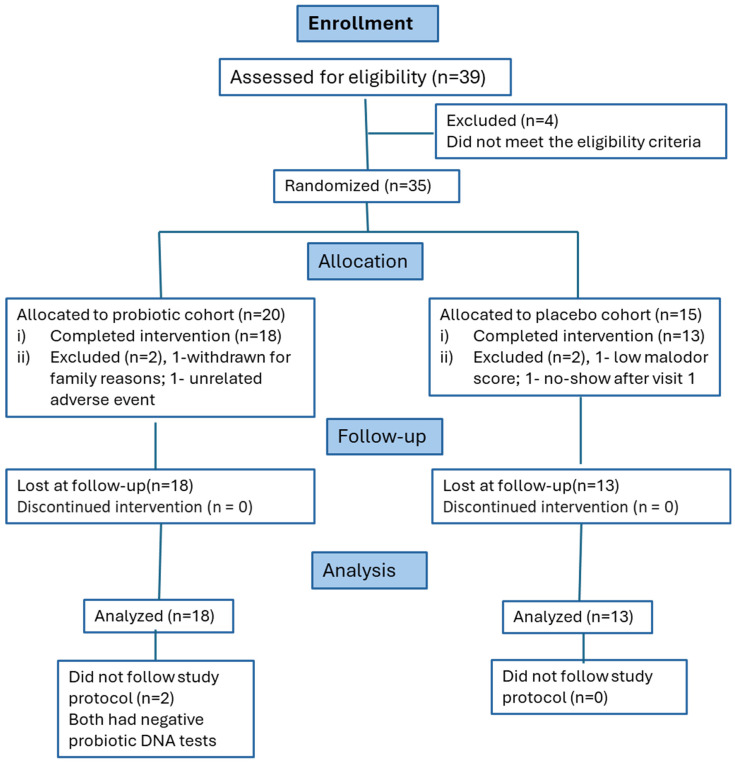
CONSORT flow diagram of participants who participated in the study, according to the CONSORT 2010 Statement.

Thirty-nine volunteers were screened, and thirty-five participants consented (Figure 3). All consenting participants were randomized into either placebo (*n* = 15) or probiotic (*n* = 20) cohorts. Thirty-one participants completed the study. Two participants were withdrawn from the probiotic cohort. One participant had an adverse event unrelated to the probiotic 3 days after enrolment, and one said they would not participate in the study due to family-related reasons. In the placebo cohort, two participants were withdrawn by the Principal Investigator due to one not meeting the eligibility criteria (a low malodor score at the consent), and the second participant being a no-show after the first visit.

Demographic characteristics are presented in Table 2. The average age was 28.1 and 27.3 years in the placebo and probiotics cohorts, respectively. The placebo cohort had slightly more males compared with the probiotic group. Cohorts were ethnically different, with the probiotic cohort having participants of White-Hispanic and Black, Non-Hispanic ethnicities. The test for equal demographic randomization showed that the differences were not statistically significant, with *p* = 0.240 for race/ethnicity, *p* = 0.727 for gender, and *p* = 0.599 for age differences. Overall, the trial participant cohort represented the Utah State population (Table 2), which is a predominantly White population with an average age of ~30 years old [3]. The percentage of Asian, White-Hispanic, and Black ethnic groups was slightly higher than that in the Utah population.

All tongue coating samples collected from two participants in the probiotic cohort were negative for probiotics’ DNA. However, data collected from these participants were used to analyze the trial results.

### 3.2. Effect of Proper Dental Hygiene and Probiotic Use on Malodor

**Figure 4:**
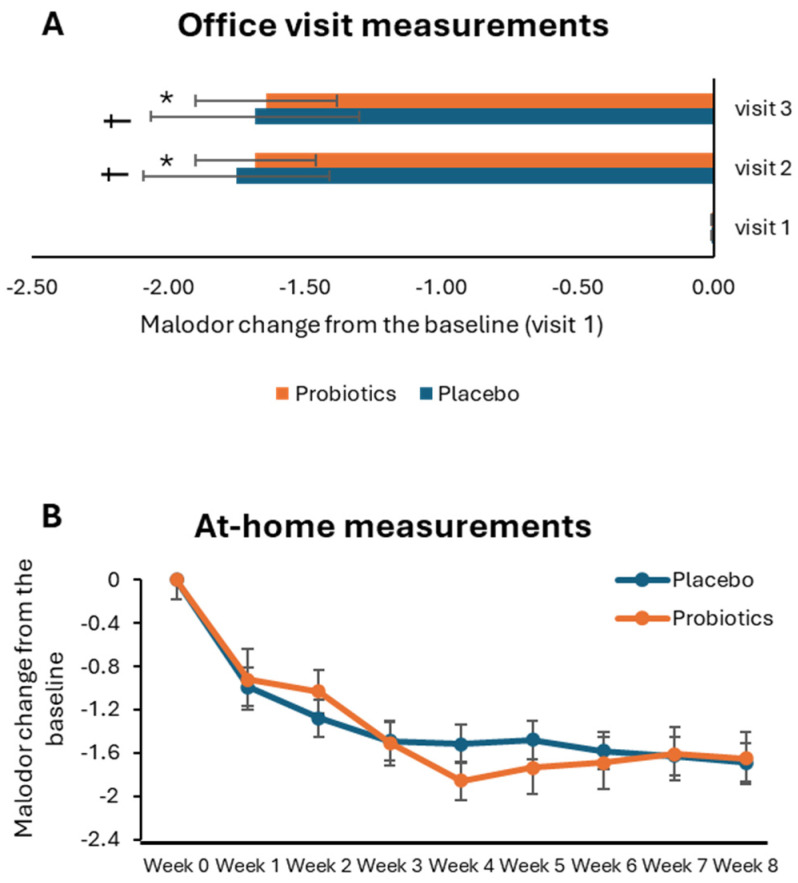
Malodor score changes. (**A**)—the measurements were performed by participants during office visits; *—the differences between the baseline measurements and visit 2 measurements in the probiotic cohort were statistically significant (*p* < 0.0001); †—the differences between the baseline measurements and visit 2 measurements in the placebo cohort were statistically significant (*p* < 0.0001); there were no statistically significant differences in the malodor reduction between the placebo and probiotic cohorts. (**B**)—The measurements were taken by participants at home.

To assess the effect of proper DH and the use of probiotics on the level of malodor, participants self-evaluated organoleptic (malodor) scores in between teeth at six positions during three office visits and weekly at home (Figure 4A,B). The baseline mean malodor levels were 3.22 and 3.28 in the placebo and probiotic cohorts, respectively. The malodor scores were reduced by more than 50% 30 days after interventions started, and the decreased scores were retained during the follow-up period (Figure 4A). The intracohort differences in the malodor scores compared to baseline were highly significant in both cohorts, with *p* values < 0.0001. However, there was no statistically significant difference in the malodor reduction between the placebo and probiotic cohorts.

At-home malodor measurements are plotted in Figure 4B. The probiotic cohort had a slightly higher decrease in malodor scores compared to the baseline by weeks 4 and 5 compared to the placebo cohort. After week 5, the malodor differences were similar in placebo and probiotics cohorts. The differences in the reductions in malodor scores between the probiotic and placebo cohorts were not significantly different.

### 3.3. Effect of Proper Dental Hygiene and Probiotic Use on Tongue Coating

To assess the effect of proper DH and probiotics on the intensity of tongue coating, we evaluated the intensity of the tongue coating on a scale of 0–2 during office visits. The results of the tongue coating evaluation are presented in Table 3. The tongue coating intensity decreased by 45% in placebo cohorts during the intervention compared to the baseline measurements (*p* = 0.011). The reduced tongue coating scores remained approximately the same level following the follow-up visit (*p* = 0.018).

In the probiotic cohort, the intensity of tongue coating decreased by 46% the intervention compared to baseline (*p* = 0.008). However, the tongue coating scores measured at the follow-up visit in this cohort increased by 47.5% compared to the intervention scores but remained lower than the baseline. The changes in the tongue coating scores between the placebo and probiotics cohorts were not statistically significant.

### 3.4. Effect of Proper Dental Hygiene and Probiotic Use on Salivary pH

Salivary pH plays a critical role in supporting oral health [20]. Saliva has a normal pH range of 6.2–7.6. The salivary pH in both cohorts was within the normal range and did not change significantly after the recommended DH and probiotics (Table 4).

### 3.5. Effect of Proper Dental Hygiene and Probiotic Use on the Probiotics’ DNA Levels in the Tongue Coating

Although the primers used to analyze probiotics’ DNA levels in the tongue coatings of participants were specific to the probiotic strains used in the study, we detected the DNA of bacterial strains similar to the strains that are part of the probiotic lozenges in the baseline tongue coating samples (Table 5). At baseline, the detectible DNA of *L. plantarum* was present in 38–39% of participants in both cohorts. The detectible *L. brevis* DNA was found in 15–16% of participants. The most frequently detected strain was *P. acidilactici,* with 67–69% of participants having detectible *P. acidilactici* DNA in their tongue coating samples.

In the probiotics group, the copy numbers of *L. plantarum* DNA levels, normalized by the 16S gene, increased 10.7 times after the 30 days of the intervention (Table 6). The placebo group showed a decrease in *L. plantarum* DNA levels after 30 days of the intervention. The *L. plantarum* DNA levels after the 30 days of intervention were significantly higher in the probiotics cohort compared to the placebo cohort (*p* = 0.047, Table 6).

The *L. brevis* DNA levels, normalized by the 16S gene, increased 36 times after the 30 days of the intervention, followed by a decrease 6-fold during follow-up in the probiotic cohort (Table 6). The *L. brevis* DNA levels were low in the placebo cohort, except for the 30-day follow-up, where we identified one outlier with 92,760 *L. brevis* copy numbers. After the removal of the outlier, the remaining samples had undetectable or very low levels of *L. brevis* DNA. The difference between the groups did not reach statistical significance.

The *P. acidilactici* DNA levels were high at baseline, with over 60% of participants showing detectable levels (Table 5). The *P. acidilactici* DNA copy numbers decreased in the placebo cohort and, as expected, increased in the probiotic cohort in tongue coating samples after 30 days of lozenge use (Table 6). The Wilcoxon rank sum test revealed no statistically significant differences between probiotic DNA levels in intervention and follow-up samples in the placebo and probiotic cohorts compared to the probiotic DNA levels at baseline (Table 6). No statistical differences were detected between probiotic DNA levels in samples collected at baseline, intervention, and follow-up visits in the placebo and probiotics cohorts.

**Figure 5:**
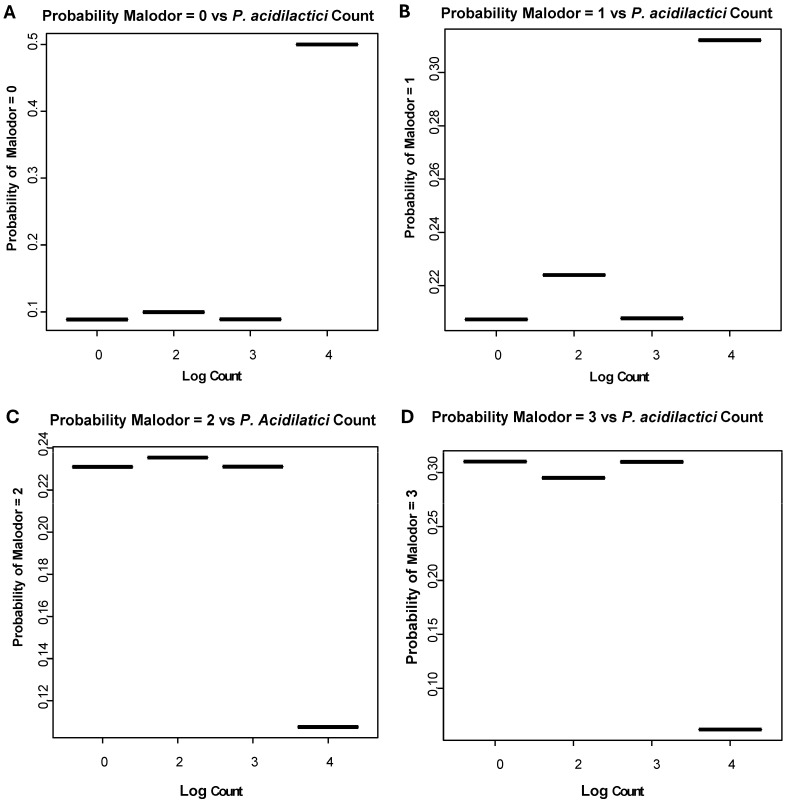
The probability of having a malodor score depends on the levels of *P. acidilactici* in the tongue coating. (**A**)—Malodor score of 0; (**B**)—malodor score of 1; (**C**)—malodor score of 2; (**D**)—malodor score of 3.

Based on the analysis of the probability of having malodor scores of 0–3, we have concluded that the malodor intensity (scores) depended on the levels of *P. acidilactici* present in the tongue coatings of the participants (Figure 5). The absence of malodor and a questionable malodor were associated with the high levels of *P. acidilactici* DNA (>1000 copies) present in the tongue coating. The relationship between malodor and the level of *P. acidilactici* was independent of visits.

### 3.6. Effect of Proper Dental Hygiene and Probiotic Use on Salivary Cytokines

The analysis of the cytokines’ levels revealed that despite selecting participants with similar malodor scores, the oral probiotic cohort had higher levels of inflammatory cytokine levels at the baseline (Table 7). These differences were not statistically different. All cytokine levels were within the levels reported by others [21].

The IL-1β was reduced gradually in both cohorts in intervention and follow-up saliva samples compared to the baseline (Table 7). Although the changes in IL-1β levels were statistically significant compared to the baseline within the cohorts compared to the baseline (*p* < 0.0001), the differences between the placebo and probiotic cohorts were not statistically significant.

The placebo cohort had an 12% increased level of IL-8 in samples collected on the 30th day of the intervention compared with the baseline levels, and the level of IL-8 decreased by 9% in the follow-up samples (Table 7). The IL-8 levels steadily declined compared to the baseline in the probiotic cohort during the study period. The changes in IL-8 levels in the intervention and follow-up samples were statistically different compared to the baseline within each cohort (*p* < 0.0001) but not different when IL-8 level changes were compared between the two cohorts.

In the placebo cohort, the levels of IL-6 and TNFα increased in samples collected on the 30th day of the intervention compared to the baseline by 47% and 19%, respectively (Table 6). During the follow-up period, the TNF-α and IL-6 levels were decreased compared to the intervention period but were above the baseline in the placebo cohort. In the probiotic cohort, TNF-α and IL-6 levels increased above the baseline on the 30th day of the intervention and fell by 33% and 72% in the follow-up samples compared to the baseline. The intracohortal changes in the TNF-α and IL-6 levels in interventional and follow-up samples compared to the baseline samples were statistically significant (*p* < 0.0001), but the differences in cytokine changes compared to the baseline between the placebo and probiotic cohorts were not statistically significant.

The levels of IL-10 have gradually decreased in salivary samples collected during intervention and follow-up periods compared to the baseline in the placebo cohort (Table 7). On the contrary, IL-10 levels were up-regulated on the 30th day of the intervention and then dropped during the follow-up period compared with the baseline in the probiotic cohort.

## 4. Discussion

A healthy oral microbiome supports oral health and may prevent and counteract oral diseases [22]. Probiotic strains of bacteria naturally present in the oral cavities of healthy individuals may contribute significantly to their oral health. Many strains of probiotics were discovered. The question arises of which strain is suitable to support oral or mental health. The strains suitable as probiotics supporting oral health should be strains that can colonize the oral cavity effectively, survive harsh oral conditions, and antagonize oral pathogens [23]. Bosch and colleagues investigated a hundred strains isolated from the oral cavity or feces healthy children [23]. Forty-six isolates belonged to the lactic acid bacteria genera; seven were considered the best candidates for improving oral health. Among them were *Lactobacillus brevis*, *Lactobacillus plantarum*, and *Pediococcus acidilactici,* which were part of the study lozenges. A recent review article summarized data on the efficacy and safety of several probiotic strains that recently became available on the US market [24]. The first candidates were *Streptococcus* spp., which were improving oral health by producing bacteriocins and hydrogen peroxide. The second group of bacteria were stains from the *Lactobacillus* genus. Among them are *Lactobacillus plantarum* and *Limosilactobacillus reuteri.* Unfortunately, no consensus exists on whether probiotics effectively prevent or treat oral diseases [22].

This pilot placebo-controlled randomized clinical study was designed to test the effectiveness of proper DH and a probiotic dietary supplement comprising freeze-dried lactic acid bacteria (*Lactobacillus brevis* KABP052, *Lactobacillus plantarum* KABP051, and *Pediococcus acidilactici* KABP053) in reducing malodor and tongue coating scores and supporting overall oral health. The study’s results indicate that proper DH alone or combined with probiotics could improve oral health, as evidenced by decreased malodor and tongue coating scores throughout the study. These results suggest that the malodor the participants reported in this study was physiological and potentially associated with not being recommended by dentists for oral hygiene or not using appropriate cleaning techniques. The mechanical removal of bacteria causing malodor is essential in reducing unpleasant odor from the mouth [25]. Flossing could be another factor responsible for reducing malodor. For example, water flossing for 12 weeks reduced the malodor scores compared to toothbrushing alone [26].

Unfortunately, there were no statistically significant differences between the placebo and probiotics cohorts in our study. This could be due to the small power of the study, but most likely, probiotics are more effective in supporting oral health when combined with prophylactic dental procedures. It has been reported that probiotics were more effective when used with clinical periodontal treatment than with the probiotic treatment alone, after which only moderate improvements in gum bleeding and probing depth were reported [14].

Dong-Suk Lee and colleagues reported that the use of *Weissella cibaria* after professional cleaning for 8 weeks decreased organoleptic scores in people with bad breath [25]. In our study, a malodor reduction was moderately associated with elevated levels of *Pediococcus acidilactici* in the tongue coatings of the participants. Another clinical study reported that high levels of *L. brevis* were associated with a reduced plaque index in patients with healthy gingiva or mild gingivitis [27].

Individuals with halitosis saw a significant reduction in volatile sulfur compounds after 1 week of treatment with *S. salivarius* K12 and chlorhexidine volatile sulfur compounds [28]. The authors suggested that the effectiveness of *S. salivarius* K12 may be due to the ability of this strain to produce bacteriocin. The efficacy of probiotic strains may be associated with their ability to produce antimicrobial peptides or antagonize pathogenic bacteria growth. The systematic review supports the use of probiotics as an adjunct therapy for non-surgical treatment of periodontal diseases [29]. However, the recent meta-analysis suggests that probiotics may be able to treat halitosis and periodontitis, but a diversity of regimens and probiotic strains does not allow for credible conclusions [30].

There are several reports demonstrating the benefits of probiotics in people with no malodor or people who had physiological halitosis. The use of probiotics containing *Lactobacillus rhamnosus* GG and *Bifidobacterium animalis* subsp. lactis BB-12 by healthy volunteers reduced the plaque index and the gingival index; however, the probiotics did not improve the microbiome of participants [31]. Individuals with halitosis consuming *L. reuteri* DSM 17,938 and *L. reuteri* ATCC PTA 5289 reported significantly lower organoleptic scores after 14 days of probiotic use [32]. People with halitosis using *L. brevis* CD2 for 14 days improved only their tongue coating scores [33].

Biofilm and pathogenic microorganism activity in the oral cavity (e.g., teeth and tongue coating) are the common causes of malodor [34]. Probiotics disrupt biofilm formation and prevent quorum sensing and the survival of bacteria forming biofilms [35].

The effectiveness of probiotics was studied in clinical settings, for example, as an adjuvant treatment after oral surgery in patients with caries or periodontal diseases [2,14,22,36]. It appears that the use of probiotics as an adjunct therapy for periodontal diseases may combat dysbiosis and modulate the immune response associated with gingivitis, periodontitis, and others [37]. Other investors used probiotic blends to treat gingivitis or reduce postoperative pain after molar extraction [2,36]. Eduardo Montero and colleagues concluded that the treatment with the probiotic blend did not significantly reduce the gingival index but reduced operative site inflammation [36]. The systematic review evaluated the impact of probiotic use on maintaining oral health in patients during a fixed orthodontic treatment [38]. It has been concluded that the use of probiotics may reduce levels of streptococcal species, causing white spot lesions in this patient.

The significant variation in the malodor scores between participants within cohorts in our study led us to think that participants may have different levels of natural probiotics in their oral cavities detected by primers designed for the detection of the study probiotic strains or that some probiotic cohort participants did not take the probiotics. Additionally, we wanted to know how long the probiotics given to the participants would be detectable in the tongue coating after participants stopped using probiotic lozenges. We found that bacterial strains similar to the *L. brevis*, *L. plantarum*, and *P. acidilactici* strains present in the lozenges are part of the microbiome of some study participants. Of note, the primers were specifically designed to detect the probiotic strains used in the study. We discovered that bacterial strains present in the tongue coatings of some participants were amplified with these primers. Our finding agrees with data from José Nart and colleagues detecting *L. brevis* and *L. plantarum* naturally present in the oral microbiota [27]. One of our findings is quite alarming. We found that over 60% of participants had *P. acidilactici* in the tongue coatings at baseline. The low malodor scores were associated with the higher levels of *P. acidilactici* regardless of the study period. *P. acidilactici* is naturally present in many fermented foods [39]. Participants might consume fermented food containing *P. acidilactici.* Therefore, the changes in malodor, tongue coating, or other parameters may not be directly attributed to the effect of the *P. acidilactici* strain in the probiotic lozenges. Feasibly, when new trials are designed, researchers need to use as an exclusion criterion—avoiding fermented food that might contain *P. acidilactici.*

Following the analysis of the levels of probiotics in the tongue coatings, we observed a reduction in the probiotic DNA levels after the introduction of proper DH, which could be potentially explained by the mechanical removal of the bacteria naturally present in the tongue coating. As we expected, the levels of all three probiotic strains have increased in the probiotic cohort after 30 days of probiotic use. The levels of probiotics decreased in the follow-up period, suggesting that individuals with malodor should use probiotics continuously to have an effect.

The analysis of probiotic DNA levels in the tongue coating could also help to identify participants who were not using probiotics. In our study, we identified two participants whose samples were negative for all strains of the probiotic formulation.

Oral health is closely associated with cytokine production by immune cells present in the oral cavity. Increased levels of inflammatory cytokines, primarily IL-1α and IL-6, may be associated with periodontal, oncological, and infectious diseases [8,40]. IL-1β is strongly associated with dysbiosis, resulting in oral inflammation in patients with gingivitis and periodontal disease [41]. In orthodontic patients, salivary IL-1β levels were moderately associated with total aerobic and anaerobic bacteria counts [42]. L-8 levels were elevated in symptomatic patients with periodontal disease [43]. L-8 is a well-known chemoattractant of neutrophils and an inducer of inflammatory processes [44]. Fibroblasts, endothelial and phagocytic cells present in the oral cavity, produce this cytokine.

In our study, proper DH and, specifically, probiotics decreased IL-1β levels by the end of the study. IL-8 levels increased during the intervention and decreased during the follow-up period in participants of the placebo cohort. In contrast, the co-administration of the probiotic reduced IL-8 production during the intervention and follow-up periods. Salivary TNF-α and IL-6 levels increased during the intervention period and decreased following the follow-up period. TNF-a, IL-6, and IL-8 can be potentially explained by the microtrauma resulting from the initial mechanical teeth brushing and flossing. The healing led to a decrease in the cytokine levels in this cohort. The gradual decrease in IL-8 production may suggest that these probiotics regulate the activity of immune cells producing IL-8. The IL-10 levels were slightly decreased in the placebo cohort on day 30 after intervention initiation. In contrast, the IL-10 levels were elevated in the probiotic cohort on the 30th day of the intervention. IL-10 is a potent anti-inflammatory cytokine that plays an important role in limiting the duration and extent of inflammation and, therefore, the damage to the host tissues [45,46]. These data might suggest that the addition of probiotics stimulates anti-inflammatory responses.

It has been reported that the treatment with *L. brevis* elevated levels of γ-aminobutyric acid (GABA) [2]. GABA is known for its ability to inhibit neurotransmitter signaling in the central nervous system [47], which can explain the reduction in pain reported by Ferrés-Amat and colleagues [2]. The latest research suggests that GABA also possesses anti-inflammatory properties [47].

The analysis of the feasibility of this pilot trial to conduct a potential randomized clinical trial revealed good enrolment, participant retention, and relatively good compliance with the protocol. The study coordinators approached 39 potential participants, and ~10% were not eligible (see Figure 3). Eighty-six to ninety percent of enrolled participants completed the study. We did not identify any participants who would not comply with the study protocol by reviewing lozenge blisters at office visit 2. However, the probiotic DNA analysis showed that all three probiotic strains were not detected in all samples of two participants from the probiotic cohort. This might suggest that they did not use probiotics or mouthwash during the study. This fact implies that the measurement of probiotic DNA in participants’ samples (e.g., salivary or fecal samples) could give an additional test of participants’ compliance in the probiotic trials. The primers designed to detect study probiotic strains also detected similar strains naturally present in some participants’ tongue coating, suggesting that more specific primers should be designed. We feel that this can be an impractical and challenging task. Sixty percent of participants in this trial were positive for *P. acidilactici* at the baseline. *P. acidilactici* is present in several fermented foods. We suggest adding recommendations to the future trial protocol to avoid foods during the study. Lastly, one of the outcomes of this pilot trial (salivary pH) was not helpful in determining the effects of either DH or probiotics and should be excluded from future studies.

While the changes in all trial outcomes following the intervention and follow-up periods of the study were highly significant compared to the baseline in each cohort, unfortunately, the differences between cohorts were not statistically significant. Highly variable results and the absence of statistical significance between the two study cohorts may be explained by several factors. One is the absence of professional dental cleaning for more than 1 month. Some participants may not have had dental cleaning for more than 6–12 months since the study was conducted during the COVID-19 pandemic. Many studies suggest that professional dental cleaning followed by probiotics may significantly improve oral health.

If the primary endpoint would be the intensity of malodor, based on the results of the pilot study, we determine that the sample size for the full-size randomized clinical trial should be 196, with 98 in the placebo and 98 in the probiotic cohorts with a two-sided type 1 error = 0.05, power = 0.9, sigma = 1.01 and a drop rate = 10%, and an anticipated clinical effect of a −0.5 unit of malodor change from baseline.

## 5. Conclusions

This pilot study demonstrated that proper DH and probiotics could improve oral health, evidenced by decreased malodor and tongue coating scores. A malodor reduction was moderately associated with elevated levels of *Pediococcus acidilactici* present in tongue coatings. The DNA levels of all probiotic strains were higher in the probiotic cohort after 30 days of the intervention. Probiotics addition led to an increase in salivary IL-10 levels during the intervention and a more substantial decrease in salivary IL-8, TNF-α, and IL-6 levels by the end of the study.

Based on the results of our study and previously reported clinical trials, consumers wishing to improve oral health and reduce oral malodor may benefit from practicing proper DH, regular professional dental cleaning, and the use of the probiotic blend.

More clinical studies with unified study protocols are needed to confirm the usefulness of probiotics as remedies supporting overall oral health or as an adjunct therapy for treating periodontal diseases. The study should be potentially designed with the addition of the exclusion criteria or the recommendation to refrain from eating fermented food during the study.

## Figures and Tables

**Table 1:** Calculation of the DNA copy numbers per 1 μL of the extracted sample.

Analyte	Copies of DNA per μL of the Extracted Sample
*L. plantarum*	X = 10^((CT-42.994)/−2.7788)
*L. brevis*	X = 10^((CT-40.775)/−1.7513)
*P. acidilactici*	X = 10^((CT-48.583)/−3.91)
16S	X = 10^((CT-38.939)/−2.4257)

**Table 2:** Demographics of the study participants.

	Placebo Cohort, *n* = 13	Probiotic Cohort, *n* = 18	Total Number of Participants (%) *n* = 31
**Race/ethnicity, *n* (%)**			
White-Non-Hispanic	9 (69%)	9 (50%)	18 (58%)
Asian	4 (31%)	4 (22%)	8 (26%)
White-Hispanic	0 (0%)	4 (22%)	4 (13%)
Black, Non-Hispanic	0 (0%)	1 (6%)	1 (3%)
**Gender, *n* (%)**			
Female	7 (54%)	11 (61%)	16 (52%)
Male	6 (46%)	7 (39%)	13 (48%)
**Age**			
Mean + SD	28.1 + 3.5	27.3 + 4.9	N/A *

* N/A—not applicable.

**Table 3:** Tongue coating measurements.

	Placebo Cohort, *n* = 13	Probiotic Cohort, *n* = 18
**Visit 1 (baseline)**		
Mean + SE	0.75 + 0.09	0.72 + 0.12
**Visit 2 (30 days post lozenge’s use)**		
Mean + SE	0.39 + 0.12	0.40 + 0.09
**Visit 3 (30 days follow-up)**		
Mean + SE	0.42 + 0.13	0.59 + 0.08

**Table 4:** Salivary pH measurements.

	Placebo Cohort, *n* = 13	Probiotic Cohort, *n* = 18
Mean + SE	6.83 + 0.07	6.76 + 0.08
Mean + SE	6.75 + 0.06	6.81 + 0.06
Mean + SE	6.73 +0.06	6.66 + 0.06

**Table 5:** Number of participants with detectible probiotic levels at baseline.

Cohorts	*L. plantarum*	*L. brevis*	*P. acidilactici*
Placebo, *n* = 13	5	2	9
Probiotics, *n* = 18	7	3	12

**Table 6:** The number of probiotic bacteria DNA copies per 100,000 of 16S gene in 1 μL of extracted material.

	Placebo Cohort, *n* = 13	Probiotic Cohort, *n* = 18	*p* Value
***L. plantarum***			
Baseline			
Mean + SE	197,482 + 195,685	999 + 681	1
30 days intervention			
Mean + SE	240 + 257 *	10,681 + 7883 ^†^	0.047
30 days follow-up			
Mean + SE	5081 + 3775 *	412 + 393 ^†^	0.466
***L. brevis***			
Baseline			
Mean + SE	7 + 4	737 + 709	1
30 days intervention			
Mean + SE	14 + 9 *	26,708 + 26,335 ^†^	0.304
30 days follow-up			
Mean + SE	77,301 + 77,300 *	125 + 90 ^†^	0.079
***P. acidilactici***			
Baseline			
Mean + SE	839,724 + 795,780	141,452 + 125,536	1
30 days intervention			
Mean + SE	2933 + 2917 *	36,180 + 288,769 ^†^	0.584
30 days follow-up			
Mean + SE	807,375 + 470,904 *	21,348 + 20,908 ^†^	0.823

* Differences between probiotic DNA levels in intervention and follow-up samples in the placebo cohort were not statistically different compared to the probiotic DNA levels at baseline (*p* = 0.46–0.88). ^†^ Differences between probiotic DNA levels in intervention and follow-up samples in the probiotic cohort were not statistically different compared to the probiotic DNA levels at baseline (*p* = 0.06–0.99).

**Table 7:** Salivary cytokine levels of the study participants.

Cytokines	Placebo Cohort, *n* = 13	Probiotic Cohort, *n* = 18	*p* Values
**IL-1β (pg/mL)**			
Baseline			
Mean + SE, pg/mL	298.35 + 93.37	371.69 + 115.81	0.564
30 days intervention			
Mean + SE, pg/mL	248.17 + 52.84 *	320.06 + 88.23 *	0.554
30 days follow-up			
Mean + SE, pg/mL	159.80 + 43.09 *	271.75 + 79.69 *	0.1044
**IL-8 (pg/mL)**			
Baseline			
Mean + SE, pg/mL	1371.30 + 308.07	2295.15 + 386.26	0.081
30 days intervention			
Mean + SE, pg/mL	1531.58 + 238.46 *	2103.40 + 378.26 *	0.671
30 days follow-up			
Mean + SE, pg/mL	1253.64 + 287.87 *	1602.88 + 421.08 *	0.755
**TNF-α (pg/mL)**			
Baseline			
Mean + SE, pg/mL	5.15 + 1.17	10.88 + 7.85	0.086
30 days intervention			
Mean + SE, pg/mL	6.11 + 1.18 *	11.66 + 2.87 *	0.6884
30 days follow-up			
Mean + SE, pg/mL	5.21 + 1.04 *	7.33 + 1.93 *	0.989
**IL-6 (pg/mL)**			
Baseline			
Mean + SE, pg/mL	5.46 + 0.79	32.34 + 21.82	0.271
30 days intervention			
Mean + SE, pg/mL	8.05 + 1.93 *	39.96 + 27.94 *	0.9207
30 days follow-up			
Mean + SE, pg/mL	6.35 + 1.22 *	9.07 + 2.59 *	0.9726
**IL-10 (pg/mL)**			
Baseline			
Mean + SE, pg/mL	1.61 + 0.56	2.76 + 0.73	0.224
30 days intervention			
Mean + SE, pg/mL	1.40 + 0.19 *	3.16 + 0.84 *	0.772
30 days follow-up			
Mean + SE, pg/mL	1.50 + 0.49 *	2.02 + 0.62 *	0.863

* Differences between levels of cytokines in intervention and follow-up samples were statistically significant compared to the baseline (*p* < 0.0001).

## Data Availability

All data are presented in the text of the paper and are available upon reasonable request from the corresponding author.
